# Supplementary material for: Distinct sensorimotor encoding in tuft dendrites and somata associated with action, correction, and learning
Source: bioRxiv. 2026 May 7:2026.05.06.722323. Preprint. [Version 1] doi: 10.64898/2026.05.06.722323 (PMC13174336; doi:10.64898/2026.05.06.722323)
Supplement: Supplementary file 1 [file NIHPP2026.05.06.722323v1-supplement-1.pdf]

bioRxiv preprint doi: https://doi.org/10.64898/2026.05.06.722323; this version posted May 7, 2026. The copyright holder for this preprint (which was not certified by peer review) is the author/funder, who has granted bioRxiv a license to display the preprint in perpetuity. It is made available under aCC-BY 4.0 International license.

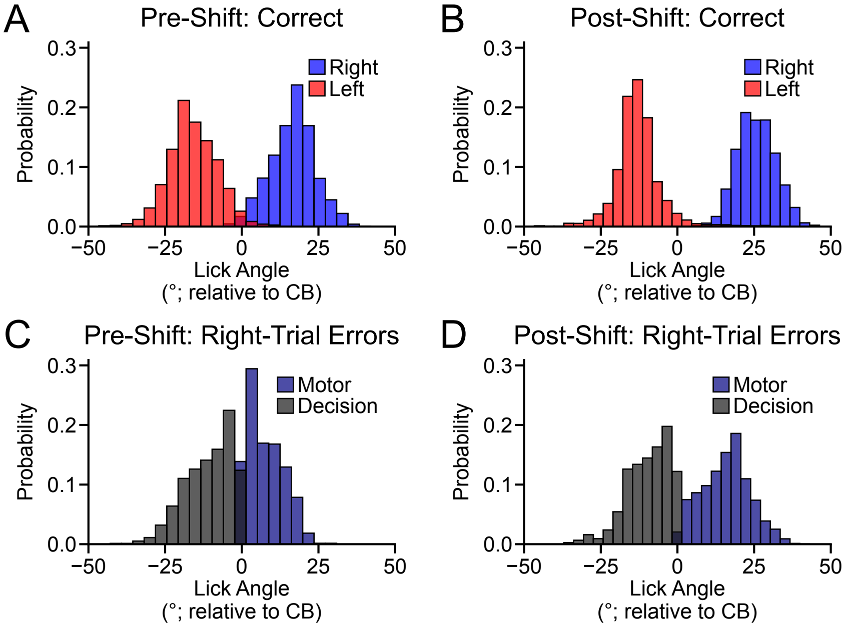

Figure 1—figure supplement 1. Tongue exit angles change after the port shift to avoid the obstructing lickport. (A) Empirical probability distributions for pre-shift correct trial types across all animals (pre-shift Correct Right $N = 10610$; pre-shift Correct Left $N = 9302$). (B) Distributions of post-shift correct trials (post-shift Correct Right $N = 6568$; post-shift Correct Left $N = 4717$). (C) Distributions of pre-shift right error trials (pre-shift Motor Error Right $N = 649$; pre-shift Decision Error Right $N = 1534$). The distribution of all pre-shift error exit angles was unimodal ($\Delta\mathrm{BIC} = -28.3$, Gaussian mixture model comparison, see Methods for details). (D) Distributions of post-shift right error trials (post-shift Motor Error Right $N = 4010$; post-shift Decision Error Right $N = 754$). The distribution of post-shift error exit angles was bimodal ($\Delta\mathrm{BIC} = 675.5$).

bioRxiv preprint doi: https://doi.org/10.64898/2026.05.06.722323; this version posted May 7, 2026. The copyright holder for this preprint (which was not certified by peer review) is the author/funder, who has granted bioRxiv a license to display the preprint in perpetuity. It is made available under aCC-BY 4.0 International license.

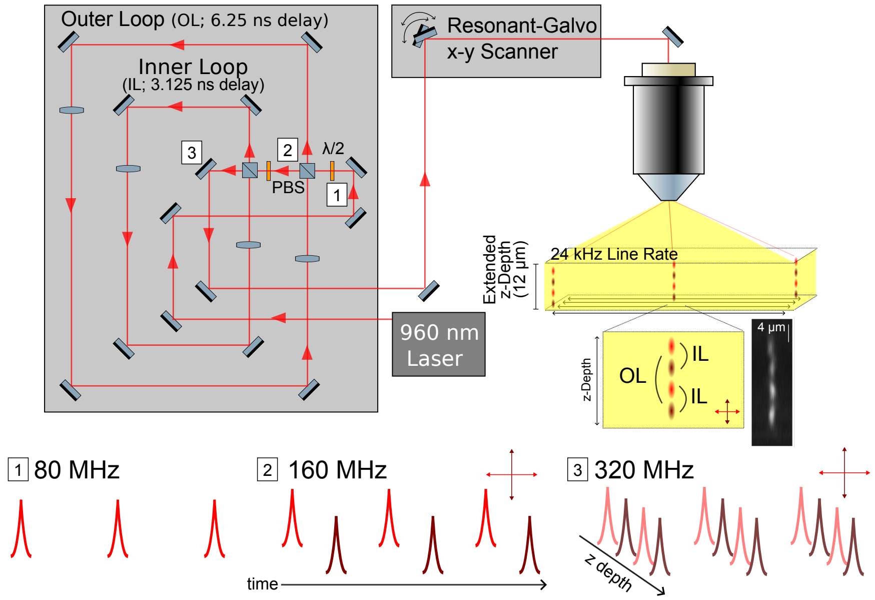

Figure 2—figure supplement 1. Optical design of module for extended axial 2P imaging. A linearly polarized 80 MHz laser pulse train (1) was split into polarization-dependent paths *via* a polarizing beam splitter (PBS), where a half-wave plate ($\lambda/2$) controls the distribution of energy to each path. The *S*-polarized component incurred a 6.25 ns delay along the *Outer Loop* with respect to the un-delayed *P*-polarized component, which was then recombined using the same PBS to form a 160 MHz pulse train with alternating polarization (2). Next, the global polarization of the 160 MHz pulse train was rotated such that the *S*-polarized components of each orthogonally polarized element of the 160 MHz pulse train incurred a delay of 3.125 ns in propagation along an *Inner Loop* with respect to the *P*-polarized components. Pulses were then recombined *via* a PBS resulting in a 320 MHz pulse train (3). Additionally, the *Outer Loop* and *Inner Loop* both contained 1:1 optical relays arranged to impart a variable divergence such that each unique path through the pulse splitter generated a distinct downstream shift in focal plane. Each of these paths were conjugated to the back aperture of the objective lens through a series of optical relays. A home-built single prism pulse compressor was used to compensate the average group delay dispersion of all delay paths and the rest of the microscope.

bioRxiv preprint doi: https://doi.org/10.64898/2026.05.06.722323; this version posted May 7, 2026. The copyright holder for this preprint (which was not certified by peer review) is the author/funder, who has granted bioRxiv a license to display the preprint in perpetuity. It is made available under aCC-BY 4.0 International license.

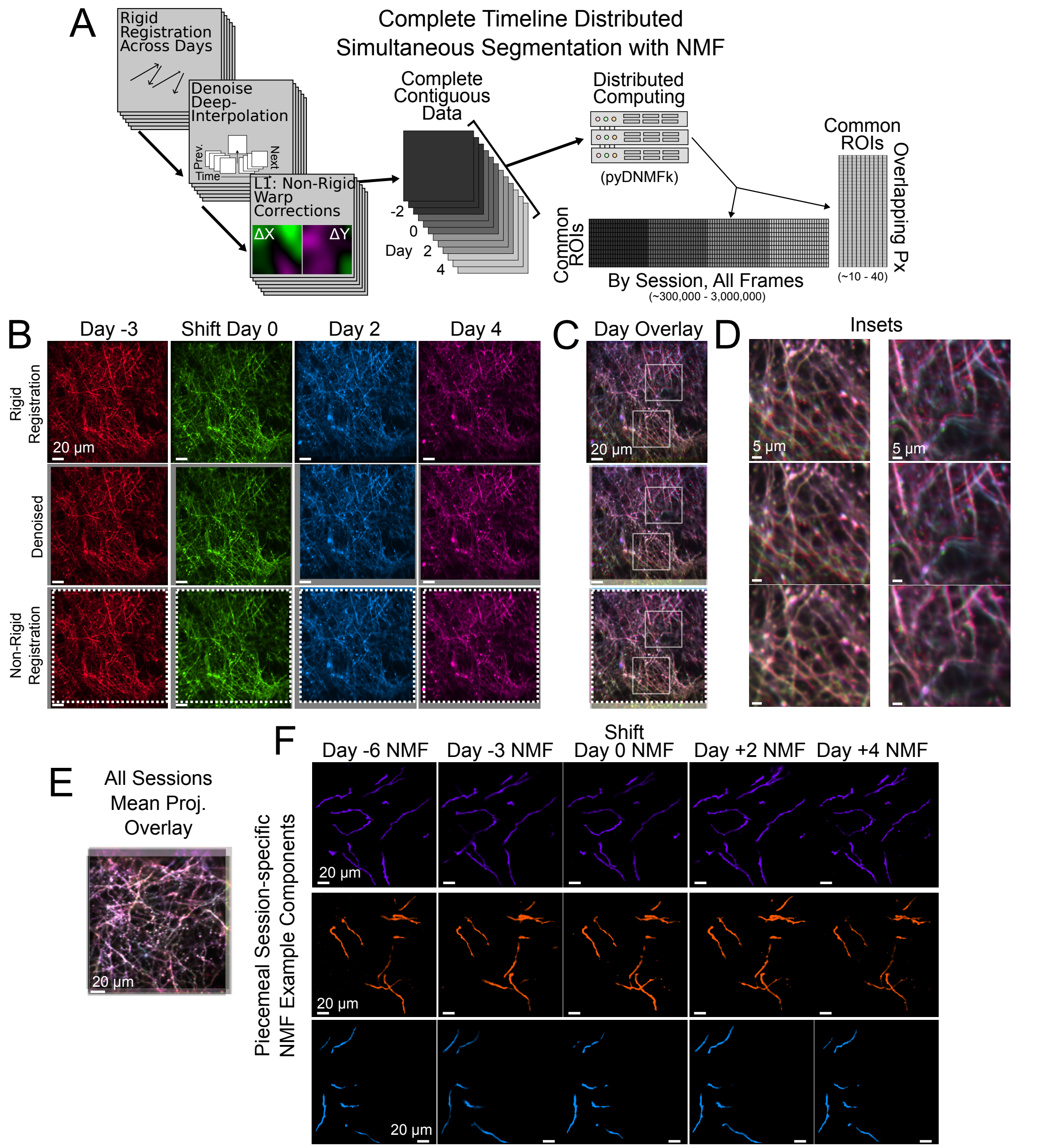

Figure 2—figure supplement 2. Image registration, denoising, and segmentation pipeline. (A) Schematic representation of the image analysis pipeline including rigid registration, denoising, non-rigid warp correction, and then complete timeline NMF. (B) Mean projection images for four dendrite imaging sessions showing the rigid registration output (top row), denoised output (middle row) and the non-rigid registration output (bottom row). Gray border areas are regions where the data were cropped prior to denoising to eliminate any regions that did not have consistent data throughout the imaging session following the rigid registration. White dotted box indicates the maximum shared region where no data was missing due to registration across all four imaging sessions. (C) Overlay of all four sessions for the corresponding rigid (top), denoised (middle) and non-rigid (bottom) corrected mean projections in (B). (D) Insets for regions in (C) showing that after rigid registration and denoising there are sometimes offsets in the positions of individual dendritic branches that must be corrected with the non-rigid registration. (E) Mean projection image overlay across all recorded sessions for an example dendritic FOV in Figure 2G. (F) Stability of NMF spatial components. Spatial components corresponding to components in Figure 2G, but calculated independently for each behavioral session.

bioRxiv preprint doi: https://doi.org/10.64898/2026.05.06.722323; this version posted May 7, 2026. The copyright holder for this preprint (which was not certified by peer review) is the author/funder, who has granted bioRxiv a license to display the preprint in perpetuity. It is made available under aCC-BY 4.0 International license.

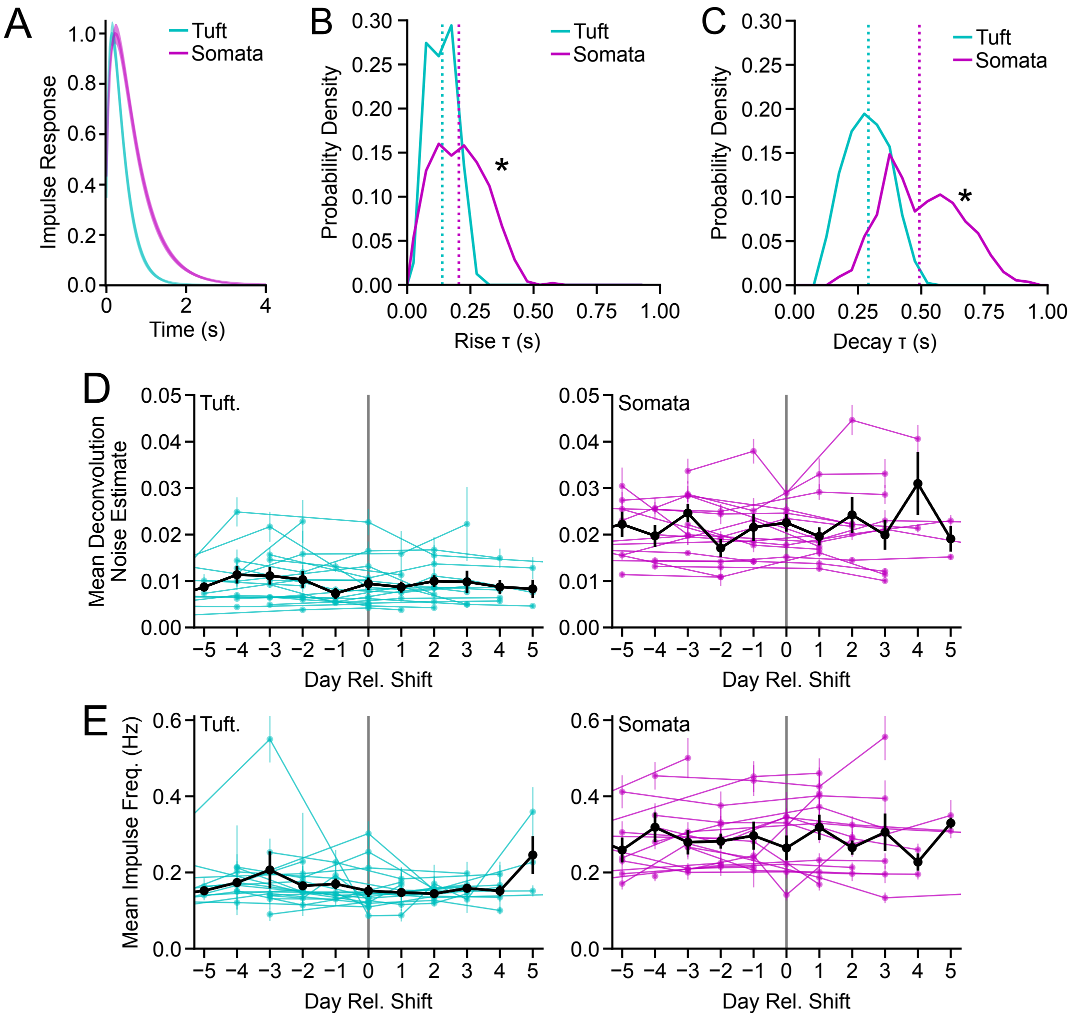

Figure 2—figure supplement 3. Estimated event kinetics, noise, and impulse rates across longitudinal 2P imaging. (A) Mean impulse response function (IRF, normalized to peak before averaging) estimated for tuft dendrites ($N = 402$ dendrites from $N = 21$ animals) and somata ($N = 528$ ROIs from $N = 20$ animals) from fits to an AR(2) model using constrained FOOPSI (***Pnevmatikakis et al., 2016***). (B) Probability densities for the rise time constant (τ) of the fits. Vertical dotted lines indicate the population median. * indicates $p < 0.05$ Kolmogorov-Smirnov (K-S) test. (C) Same as (B), but for the decay time constant (τ). (D) Standard deviation of the noise averaged across ROIs of each animal by imaging session for 5 days prior to port shift and 5 days following port shift in dendrites (left) and somata (right). Black line indicates the average noise estimate across all animals. Noise was estimated from high frequencies in the power spectral density of the trace of each ROI (***Pnevmatikakis et al., 2016***). (E) Same as (D) but for estimated impulse rates after thresholding the deconvolved traces (see Methods). Error bars: ±SEM (not bootstrapped).

bioRxiv preprint doi: https://doi.org/10.64898/2026.05.06.722323; this version posted May 7, 2026. The copyright holder for this preprint (which was not certified by peer review) is the author/funder, who has granted bioRxiv a license to display the preprint in perpetuity. It is made available under aCC-BY 4.0 International license.

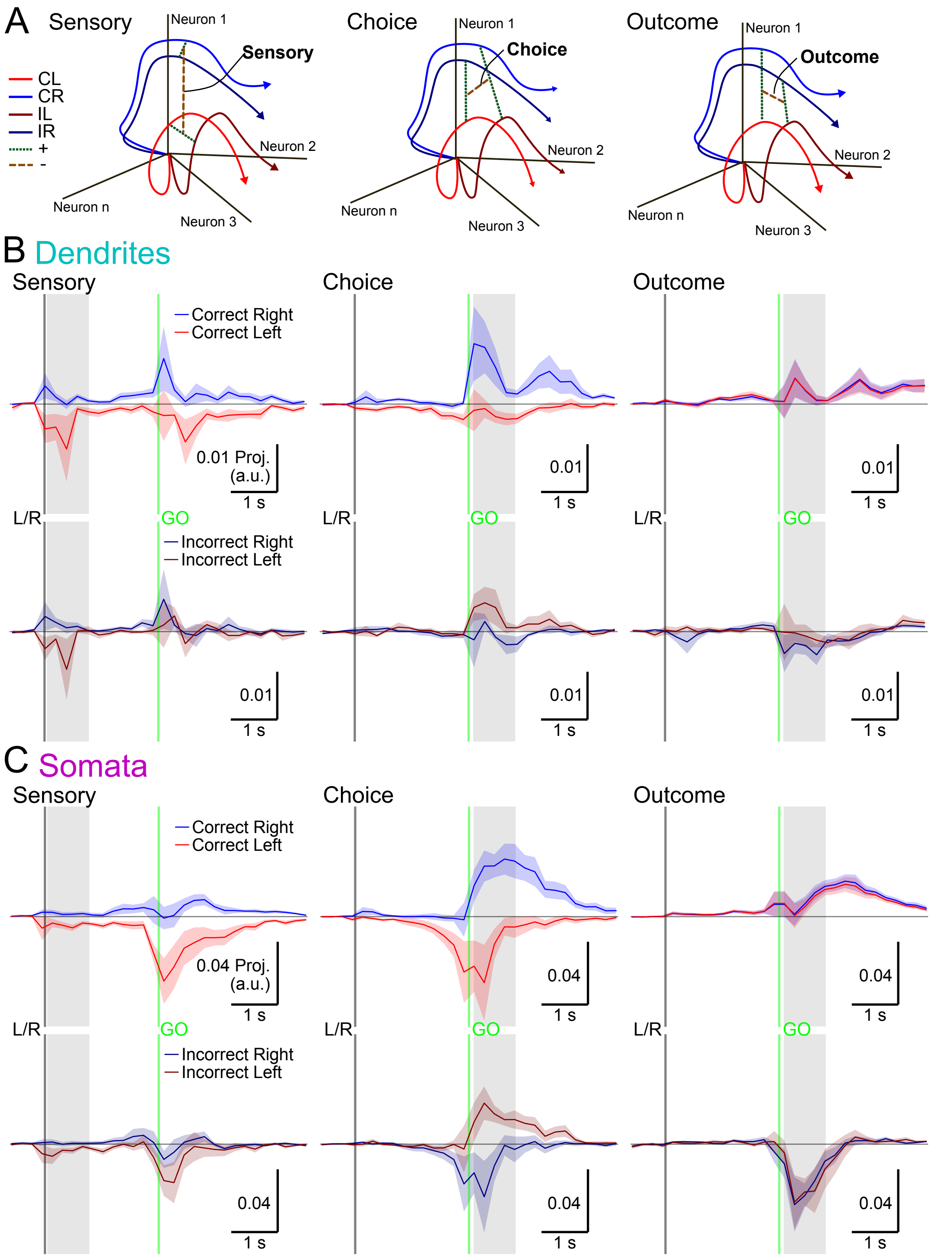

Figure 4—figure supplement 1. Mean trial-type projections along *Sensory*, *Choice*, and *Outcome* coding directions. (A) Illustration of the calculation of *Sensory*, *Choice*, and *Outcome* coding directions within activity space. For each coding direction, dotted lines indicate trial types that were first summed and the dash-dotted line indicates subsequent subtraction of the results to obtain the CD. (B) Cross-validated projections of trial-averaged activity across *Sensory*, *Choice*, and *Outcome* axes for dendrites. Correct and incorrect trial projections shown in the top and bottom of each panel, respectively. (C) Same as (B), but for all somata. Error bars: hierarchical bootstrap SEM.

bioRxiv preprint doi: https://doi.org/10.64898/2026.05.06.722323; this version posted May 7, 2026. The copyright holder for this preprint (which was not certified by peer review) is the author/funder, who has granted bioRxiv a license to display the preprint in perpetuity. It is made available under aCC-BY 4.0 International license.

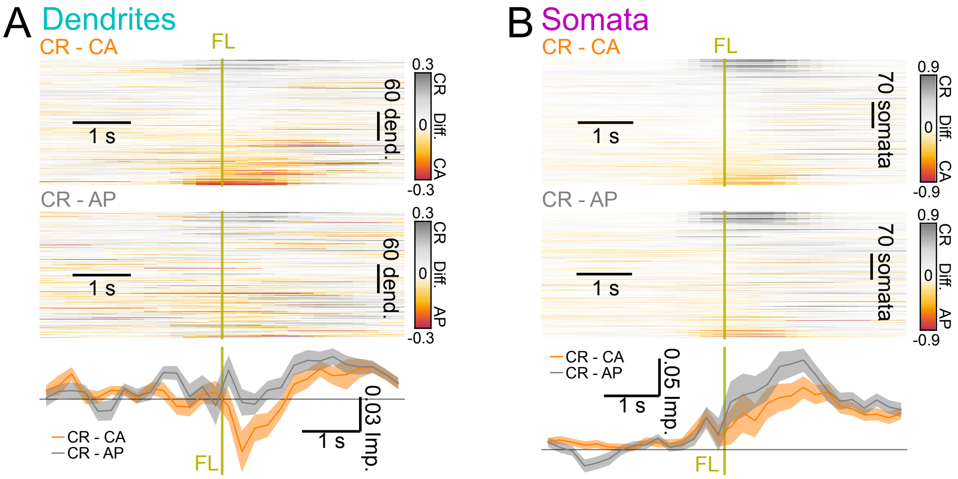

Figure 5—figure supplement 1. Differences in mean trial-aligned CR, CA, and AP activity. (A) All individual ROI differences in the mean CR minus the mean CA activity (top) or mean CR activity minus mean AP activity (bottom). ROI order is same as in Figure 5D. (B) Same as (A) for somata from Figure 5F.

bioRxiv preprint doi: https://doi.org/10.64898/2026.05.06.722323; this version posted May 7, 2026. The copyright holder for this preprint (which was not certified by peer review) is the author/funder, who has granted bioRxiv a license to display the preprint in perpetuity. It is made available under aCC-BY 4.0 International license.

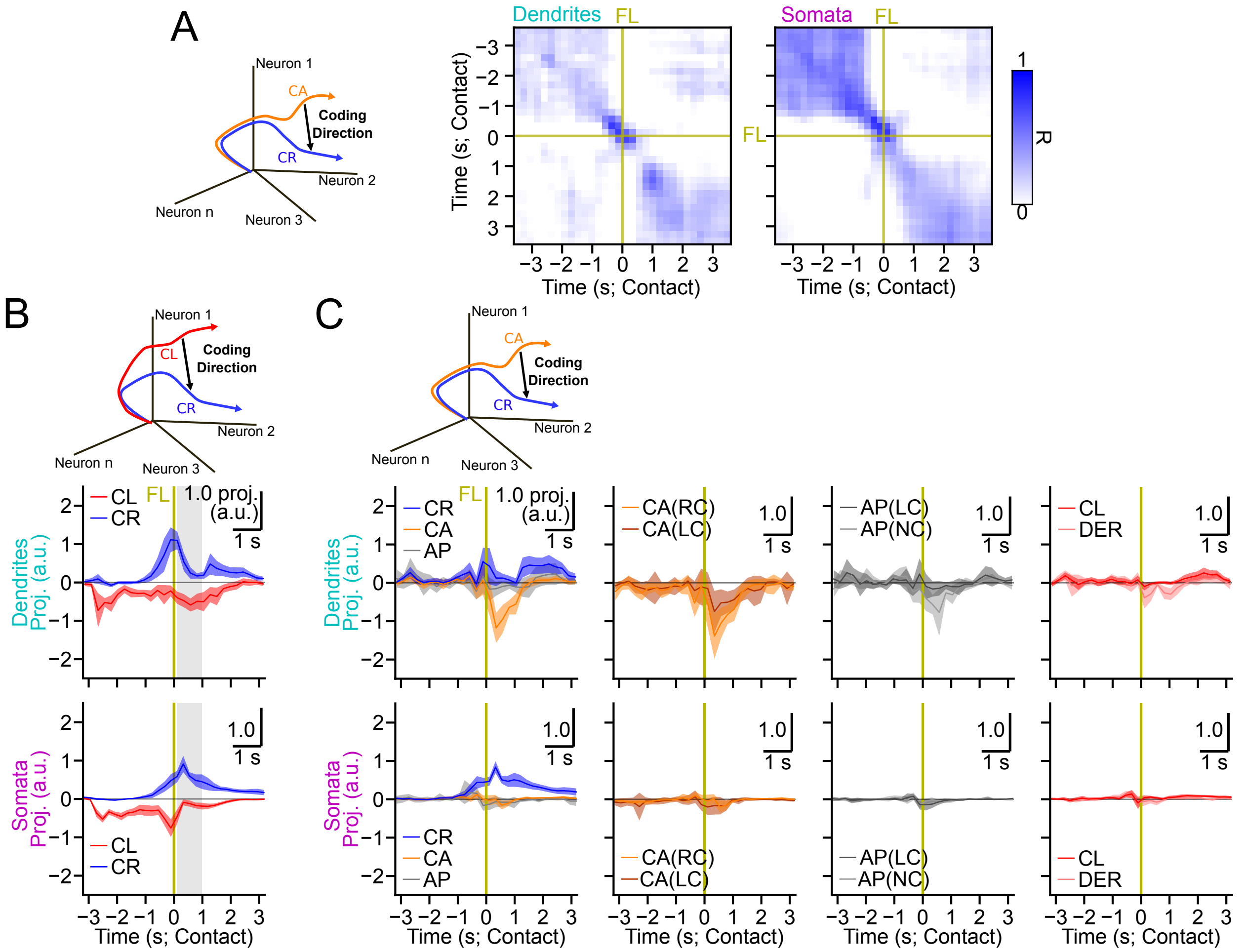

Figure 5—figure supplement 2. $CR - CA$ **code stability and additional trial-type projections.** (A) Illustration of $CR - CA$ CD calculation (left) and cross-validated correlations of $CR - CA$ CD across trial time relative to first lickport contact (FL; dark yellow line). (B) Illustration of $CR - CL$ CD calculation (top), as well as dendrite (middle) and somata (bottom) normalized cross-validated projections along the $CR - CL$ CD. Projections were normalized by division by $|CR| + |CL|$ in shaded region. (C) Illustration of $CR - CA$ CD calculation (top), as well as dendrite (middle) and somata (bottom) normalized cross-validated projections along the $CR - CA$ CD for additional trial-types. Projections were normalized by division by $|CR| + |CL|$ in shaded region of (B) to allow for direct comparisons of magnitude. Trial-type abbreviations are CR: Correct Right; CL: Correct Left; CA: Correction Attempted; AP: Abandoned Port; CA(RC): Correction Attempted (second lick made Right port Contact); CA(LC): Correction Attempted (second lick made Left port Contact); AP(LC): Abandon Port (second lick made Left port Contact); AP(NC): Abandon Port (second lick did Not make Port contact); DER: Decision Error Right.

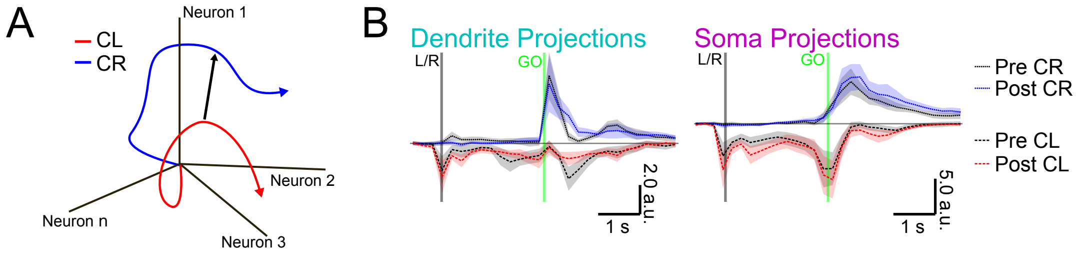

Figure 6—figure supplement 1. Trial-type projections before and after the lickport shift. (A) Schematic of the $CR - CL$ CD. (B) Cross-validated projections of CR and CL trials along the $CR - CL$ CD. Same data as used for Figure 6I,J. Shaded error: hierarchical bootstrap SEM.

bioRxiv preprint doi: https://doi.org/10.64898/2026.05.06.722323; this version posted May 7, 2026. The copyright holder for this preprint (which was not certified by peer review) is the author/funder, who has granted bioRxiv a license to display the preprint in perpetuity. It is made available under aCC-BY 4.0 International license.

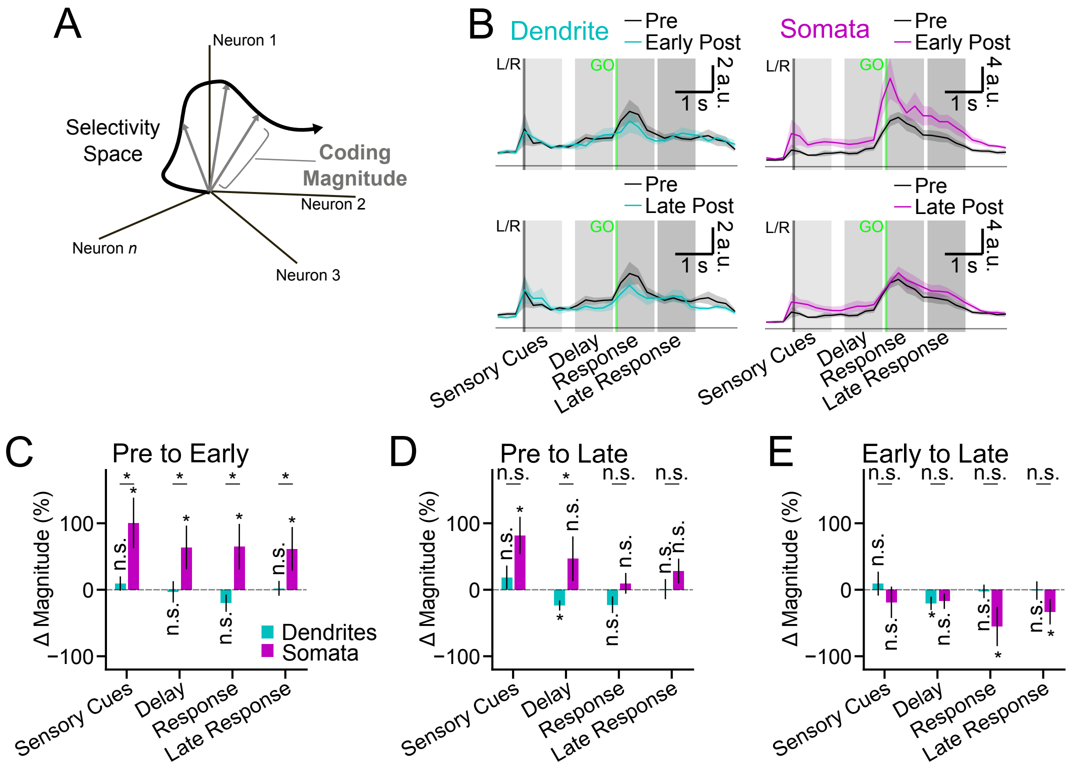

Figure 6—figure supplement 2. Selectivity CD magnitude across early and late learning. (A) Illustration of the calculation of the coding magnitude in selectivity space. (B) Coding magnitude of dendrites (left) and somata (right) during pre-shift (black), early post-shift (top row), and late post-shift (bottom row) training epochs. (C) Percent change in coding magnitude from pre-shift to early post-shift averaged within the four time windows indicated by gray rectangles in (B). (D) Same as (C), but from pre-shift to late post-shift. (E) Same as (C) but from early post-shift to late post-shift. Shaded error and error bars: hierarchical bootstrap SEM.

bioRxiv preprint doi: https://doi.org/10.64898/2026.05.06.722323; this version posted May 7, 2026. The copyright holder for this preprint (which was not certified by peer review) is the author/funder, who has granted bioRxiv a license to display the preprint in perpetuity. It is made available under aCC-BY 4.0 International license.

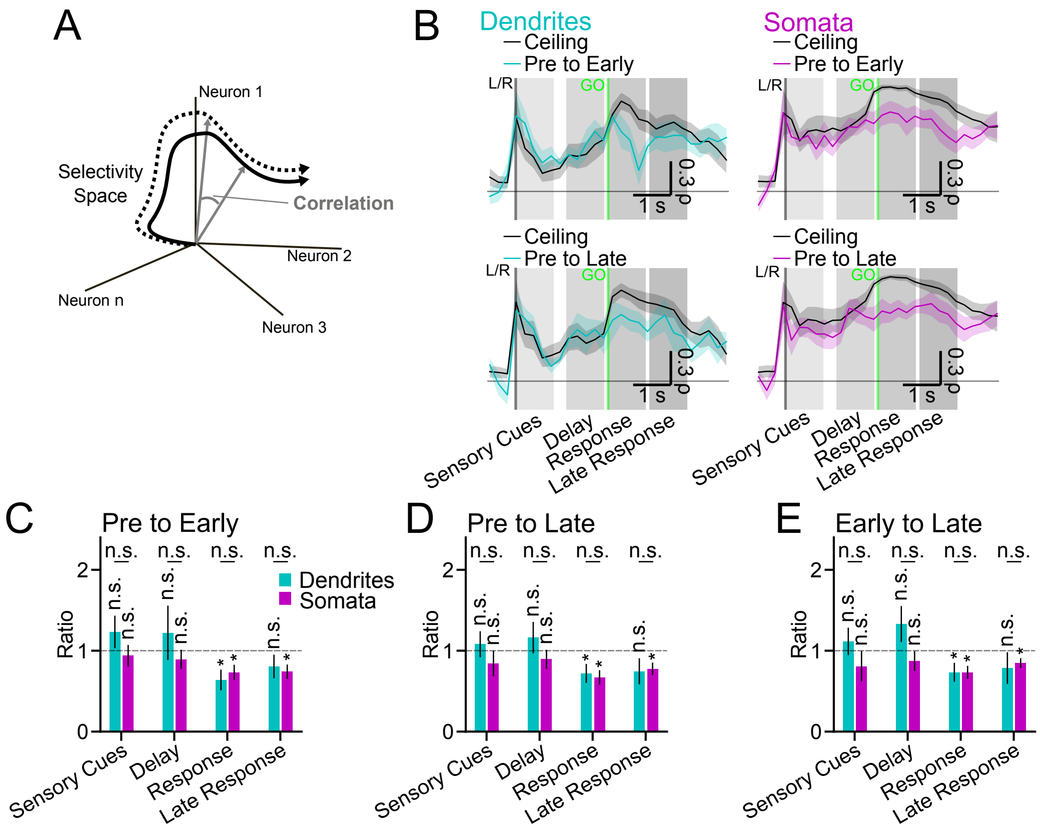

Figure 6—figure supplement 3. Selectivity CD correlations across early and late learning. (A) Illustration of correlation (cosine similarity) calculation between CDs in selectivity space. (B) Correlation between CDs calculated from pre-shift activity and CDs calculated from early post-shift activity for dendrites (top left, cyan line) and somata (top right, magenta line), as well as correlation between CDs calculated from pre-shift activity and CDs calculated from late post-shift activity for dendrites (bottom left, cyan) and somata (bottom right, magenta line). Black lines indicate the estimated maximum possible correlation ("ceiling") across the training epochs given correlations of repeated measures within each epoch (*i.e.*, the limit due to degradation by measurement noise; see Methods). (C) Ratios between the early post-shift to pre-shift correlations and the correlation ceilings in (B) averaged within the four time windows indicated by gray rectangles in (B). A ratio ≈1 indicates no change in the CD pattern across the training epochs beyond measurement noise, whereas a ratio significantly < 1 indicates significant change across the training epochs in the CD pattern. (D) Same as (C), but for correlation of late post-shift to pre-shift. (E) Same as (C), but for correlation of late post-shift to early post-shift. Shaded error and error bars: hierarchical bootstrap SEM.
